# Automated and rapid self-report of nociception in transgenic mice

**DOI:** 10.1038/s41598-020-70028-8

**Published:** 2020-08-06

**Authors:** Christopher J. Black, Anusha B. Allawala, Kiernan Bloye, Kevin N. Vanent, Muhammad M. Edhi, Carl Y. Saab, David A. Borton

**Affiliations:** 1 School of Engineering, Brown University, Providence, RI 02912 USA; 2 Department of Neuroscience, Brown University, Providence, RI 02912 USA; 3 Department of Neurosurgery, Rhode Island Hospital, Providence, RI 02903 USA; 4 Carney Institute for Brain Science, Brown University, Providence, RI 02912 USA; 5 Center for Neurorestoration and Neurotechnology, Rehabilitation R&D Service, Department of Veterans Affairs Medical Center, Providence, RI USA

**Keywords:** Sensory processing, Pain

## Abstract

There are currently no rapid, operant pain behaviors in rodents that use a self-report to directly engage higher-order brain circuitry. We have developed a pain detection assay consisting of a lick behavior in response to optogenetic activation of predominantly nociceptive peripheral afferent nerve fibers in head-restrained transgenic mice expressing ChR2 in TRPV1 containing neurons. TRPV1-ChR2-EYFP mice (n = 5) were trained to provide lick reports to the detection of light-evoked nociceptive stimulation to the hind paw. Using simultaneous video recording, we demonstrate that the learned lick behavior may prove more pertinent in investigating brain driven pain processes than the reflex behavior. Within sessions, the response bias of transgenic mice changed with respect to lick behavior but not reflex behavior. Furthermore, response similarity between the lick and reflex behaviors diverged near perceptual threshold. Our nociceptive lick-report detection assay will enable a host of investigations into the millisecond, single cell, neural dynamics underlying pain processing in the central nervous system of awake behaving animals.

## Introduction

The importance of the brain in nociception has been theorized since Descartes’ *Treatise of Man*^1^. Melzack and Wall, whose gate control theory highlights the role of the spinal cord in pain processing, acknowledged the need for the myriad, interconnected brain areas to synthesize the perception of pain^2^. Advances in human neural recording technologies have allowed scientists to test theories describing how brain circuits map onto pain^3^. Human functional MRI (fMRI) has shown that perceived pain correlates with activation of primary somatosensory cortices, anterior cingulate cortex, and other brain regions implicated in the discriminatory and affective components of pain^4,5^. Electroencephalography (EEG) and magnetoencephalography (MEG) have implicated the regulation of specific oscillatory components, for example alpha oscillations in the sensorimotor cortex, in pain perception^6^.

While techniques such as fMRI, EEG, and MEG paint a macroscopic picture of brain dynamics, these techniques cannot achieve the cellular or temporal resolution necessary for elucidating the neural circuits underlying pain perception. Genetic manipulations^7^ and in vivo neural recordings in rodents are foundational in this respect as they afford the granularity required to characterize neural circuit activity and therapeutic outcomes^8–10^. Unfortunately, the inability to compare behavioral outputs between animal and human studies presents a significant barrier for clinical translation of experimental results. Pain in humans is primarily measured using verbal self-reports, such as the visual analogue scale^11^, while pain in rodents is inferred from behavioral signals of discomfort, such as reflex behaviors^11,12^.

Reflex behaviors, like the hind paw withdrawal, are used to characterize evoked pain. Although reflex behaviors have been shown to positively correlate with self-reports of pain in humans^13^, decerebrated animal models have shown that reflex behaviors occur without the higher-order neural circuits necessary for perception^14,15^. The uncertainty of the brain’s involvement in reflex behaviors makes elucidating neural circuits difficult. This issue is further confounded by the subjectivity of scoring and the significant training demand required to perform the behavior^16^.

Self-reporting detection paradigms remove external subjectivity and inference. Rodent detection behaviors have been vital for probing visual^17^, thermal^18,19^, and tactile perception^20,21^. Rodents learn to perform specific behaviors, such as licking a water spout following stimulus detection, while concurrent neural recordings help map circuit mechanisms to the elicited behavior^22^. Currently, no self-report assay exists to examine perceptual responses to painful stimuli in animals^11^. Conventional instruments used to evoke pain lack the cellular and temporal resolution required to synchronize the millisecond time-scale neural activity to the evoked behavior. Probing the skin with von Frey mono-filaments or radiant heat activates innocuous sensory receptors^23–25^ that can confound the perception of noxious stimuli. Furthermore, reproducibility and precision of stimulus delivery is paramount to acquiring accurate behavioral results^16^. The recent implementation of peripheral optogenetics has enabled greater cellular, anatomical, and temporal precision in activating sub-populations of nociceptive afferents, but has been predominantly used to evaluate aversive or reflexive behaviors^24,26,27^. Therefore, we have developed an observer-independent, fast, lick-based detection task using peripheral optogenetic stimulation of A-delta and C-fiber afferents in transgenic mice.

## Results

### Optogenetic stimulation of TRPV1-ChR2-EYFP mice activates small diameter afferents

**Figure 1:**
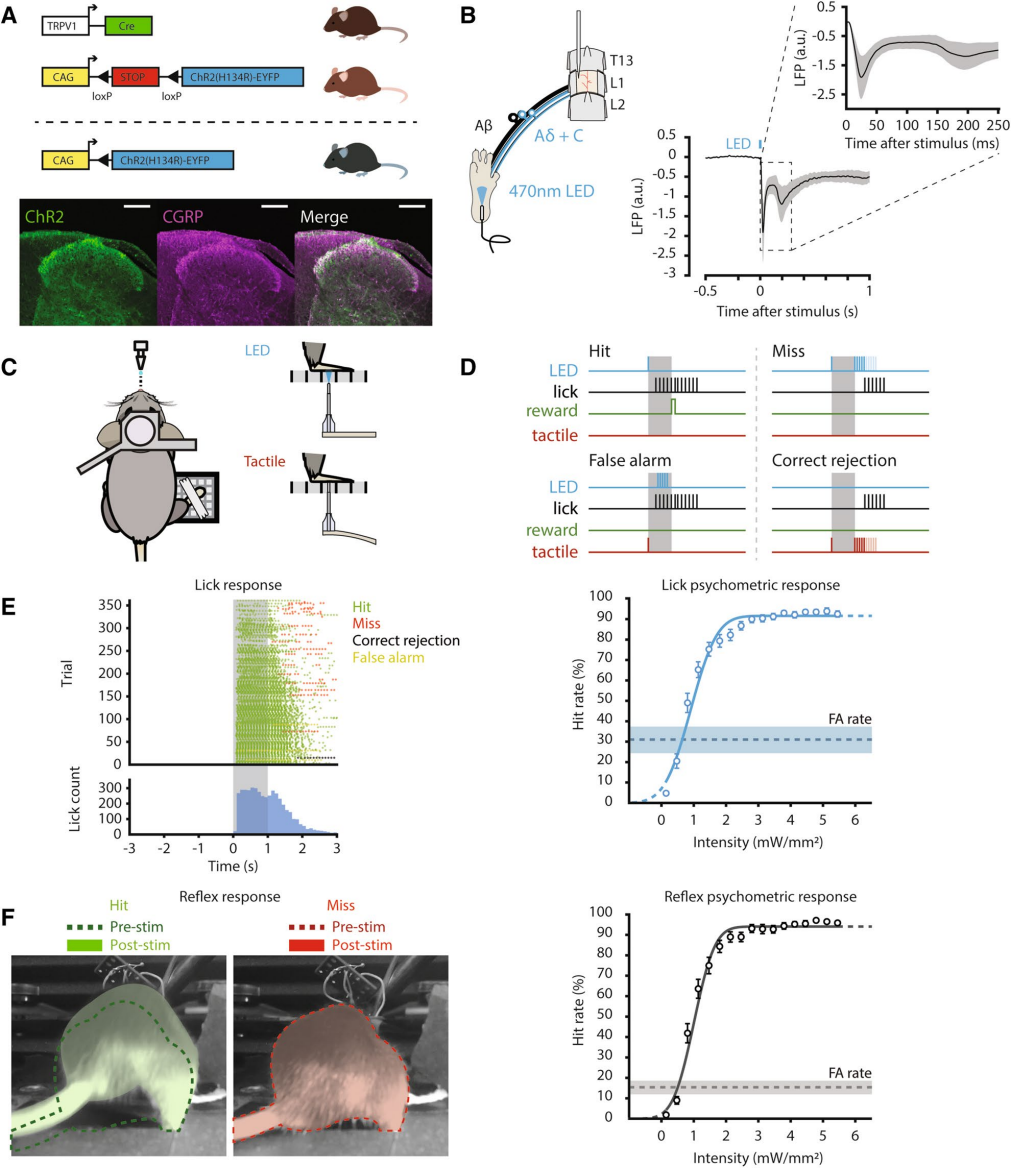
Electrophysiological characterization of TRPV1-ChR2-EYFP transgenic mice and validation of the lick behavior. (**A**) (Top) Transgen*ic mic*e expressing ChR2 in TRPV1-containing neurons were bred using a Cre driver line. (Bottom) Immunoh*istochemi*stry of spinal cord tissue in TRPV1-ChR2-EYFP mice shows overlap of ChR2 (green) with CGRP (magenta), a marker of peptidergic nociceptors; scale bar = 150 µm. (**B**) Schematic of electrophysiological recording in anesthetized mice (left). Extracellular compound action potentials (CAP) were recorded from thoraco-lumbar vertebral levels evoked by 10 ms, 470 nm LED pulses applied to the ipsilateral hind paw of TRPV1-ChR2-EYFP mice. The CAP waveform average (right, n = 6 mice, mean ± S.E.M.) shows two prominent post-stimulus peaks corresponding to A-delta (t = 20 ms) and C-fiber (t = 200 ms) afferents. (**C**) Schematic layout of behavioral setup. Mouse is head-restrained with access to a lick spout, with their right hind paw restrained over a grated floor. Stimuli are delivered through the floor to the right hind paw. (**D**) Mice receive either optogenetic (target) or tactile (catch) stimuli. Following optogenetic stimulation, licking within the 1 s post-stimulus response window was considered a ‘hit’, while the absence of licking was considered a ‘miss’. Following tactile stimulation, licking within the 1 s response window was considered a ‘false alarm’, and the absence of licking was considered a ‘correct rejection’. (**E**) Example of a lick raster and histogram (left, n = 1 session), grey shading indicates the 1 s response window, ‘hit’ responses (green) occur during the 1 s response window, with only a small percentage (5%) of ‘miss’ trials (red). False alarm responses (yellow) occurred when the mouse responded in the 1 s response window following tactile stimuli. Psychometric response (right) for lick behavior fitted with a cumulative Gaussian function, compared to false alarm (FA) rate (dotted line). (**F**) Images captured showing representative reflex behavior (left) during two different trials that elicit a response (left, shaded green) vs. no response to optogenetic stimulation (right, shaded red) as compared to baseline pre-stimulus position (dotted lines). Psychometric response (right) for reflex behavior fitted with a cumulative Gaussian function, compared to FA rate (dotted line). All psychometric responses (**E**,**F**) showing mean ± SEM, n = 40 sessions from 5 mice. Data was plotted using MATLAB R2019a (https://www.mathworks.com). The figure was created using Adobe Illustrator CC 2019 (https://www.adobe.com).

To deliver nociceptive stimuli with cellular and anatomic specificity, and temporal precision, we leveraged a well-established^24,26,28,29^ transgenic mouse line that expresses the light-sensitive ion-channel channel-rhodopsin2 (ChR2) with the enhanced yellow fluorescent protein (EYFP) tag in transient receptor potential V1 (TRPV1) containing neurons, which are responsible for peripheral transmission of noxious heat^30^. Optogenetic activation of TRPV1-ChR2-EYFP mouse glabrous skin has been shown to evoke A-delta and C-fiber mediated electrophysiological responses and nocifensive behaviors without overt tissue damage ^24,28,29^. While it has been shown that EYFP from this transgenic line predominantly co-localizes with nociceptive markers, it has also been estimated that TRPV1-lineage neurons overlap to a lesser extent (~ 8.5–24%) with non-nociceptive myelinated DRG neurons^29,31^. Therefore, in addition to confirming localization of EYFP to the peptidergic nociceptive marker calcitonin gene-related peptide (CGRP) in mouse spinal cord dorsal horn (Fig. 1A), we sought to determine the electrophysiological compound action potential (CAP) volleys of primary sensory neurons into the dorsal horn evoked by optogenetic stimulation. We recorded the extracellular field potential from the lumbosacral dorsal horn of anesthetized mice (Fig. 1B) during 470 nm optogenetic stimulation of TRPV1-ChR2-EYFP mouse glabrous skin. Afferent conduction velocities from early (1.99 ± 1.01 m/s) and late (0.20 ± 0.01 m/s) CAPs were largely in agreement with previously published values in mouse A-delta and C-fiber primary nociceptors^32^.

### TRPV1-ChR2-EYFP mice learn to self-report detection of nociceptive stimulus

A key behavioral tool in elucidating sensory perception is operant conditioning. Through training, rodents can learn to perform specific behaviors following detection of a target stimulus to receive a reward. Therefore, we used operant conditioning to train TRPV1-ChR2-EYFP mice to self-report detection of hind paw optogenetic stimulation (470 nm light). Mice were head-restrained and water restricted to incentivize lick behavior^33^, and hind paw restrained (Fig. 1C) to ensure reproducibility of stimulus delivery^18^. The behavior involved training mice to detect ‘target’ optogenetic stimuli for water reward, and avoid responding to ‘catch’ tactile stimuli (Fig. 1D). Mice displayed different affinities in training, ranging from low initial performance (< 35% correct responses) to high initial performance (> 70% correct responses) that dipped in subsequent sessions (Supplemental Figure S1). This variability in success throughout training highlights that the lick behavior is a learned task, whereas a high level of correct responses and low variability across sessions would be expected for a reflex behavior. A minimum of 7 training sessions were performed (9.8 ± 2.4 sessions) until mice displayed engagement throughout a session, which was determined by a high sensitivity (d’ > 1.0) measured across the first 250 trials and the overall session.

**Figure 2:**
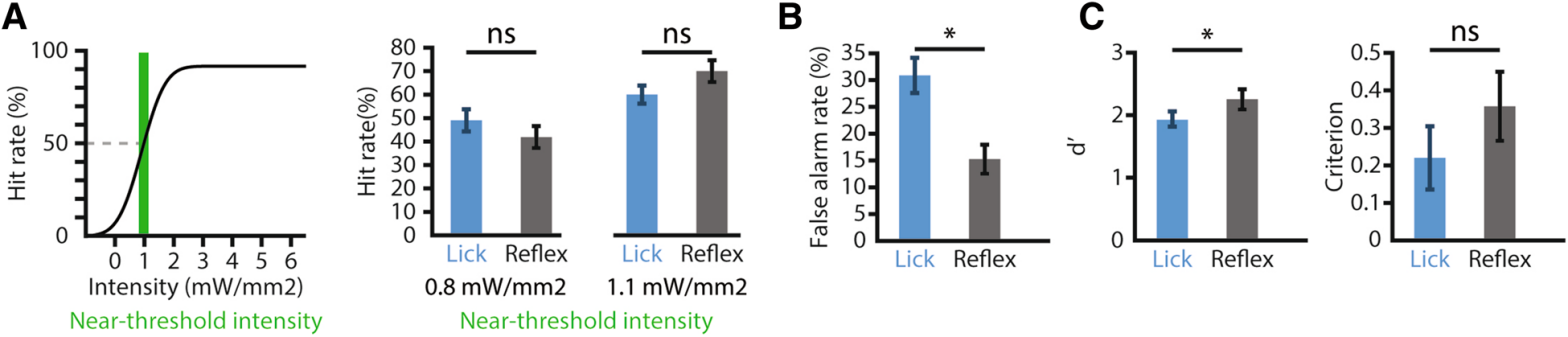
Specificity of lick behavior is distinct from withdrawal behavior. (**A**) (Left) Schematic illustration of near-threshold intensities taken from psychometric curve, (right) comparison of hit rate of lick (blue) and reflex (grey) reports at near-threshold intensities, showing no significant difference, which suggests both behaviors are comparable in detection of optogenetic stimulation. (**B**) Comparison of false alarm rate, showing the lick behavior has a significantly higher amount of false alarms than the reflex behavior, which suggests higher-order decision making plays a role in the lick response, (**C**) sensitivity (d’), and response bias (criterion) between lick (blue) and reflex (grey) reports, showing only a significant difference between behavior sensitivity, which is due largely to the significant difference in false alarm rate. Green region indicates near-threshold intensities, All results are shown as mean ± SEM (n = 40), asterisks in a-c indicate significance at *P* < 0.025 using a two-sided Wilcoxon signed rank test, b was tested for multiple comparisons with Bonferroni correction. Data was plotted using MATLAB R2019a (https://www.mathworks.com). The figure was created using Adobe Illustrator CC 2019 (https://www.adobe.com).

Mice learned to successfully lick and thereby report the detection of hind paw optogenetic stimulation, whereas no significant lick behavior was elicited during 595 nm ‘control’ stimulation between 0.14–5.45 mW/mm^2^ (Supplemental Figure S1), indicating that the lick behavior is initiated specifically by activation of ChR2-expressing afferents in the glabrous skin. Hit rates were dependent on stimulus intensity, with responses reaching an asymptotic hit rate over 90% (Fig. 1E). Furthermore, the false alarm rate was significantly lower than the hit rate of stimuli near threshold level intensity (*P* < 0.025, two-sided Wilcoxon signed rank, Bonferroni corrected). This indicates that mice learned to distinguish between nociceptive and tactile stimuli and to reliably report the detection of nociceptive stimuli specifically via lick behavior.

**Figure 3:**
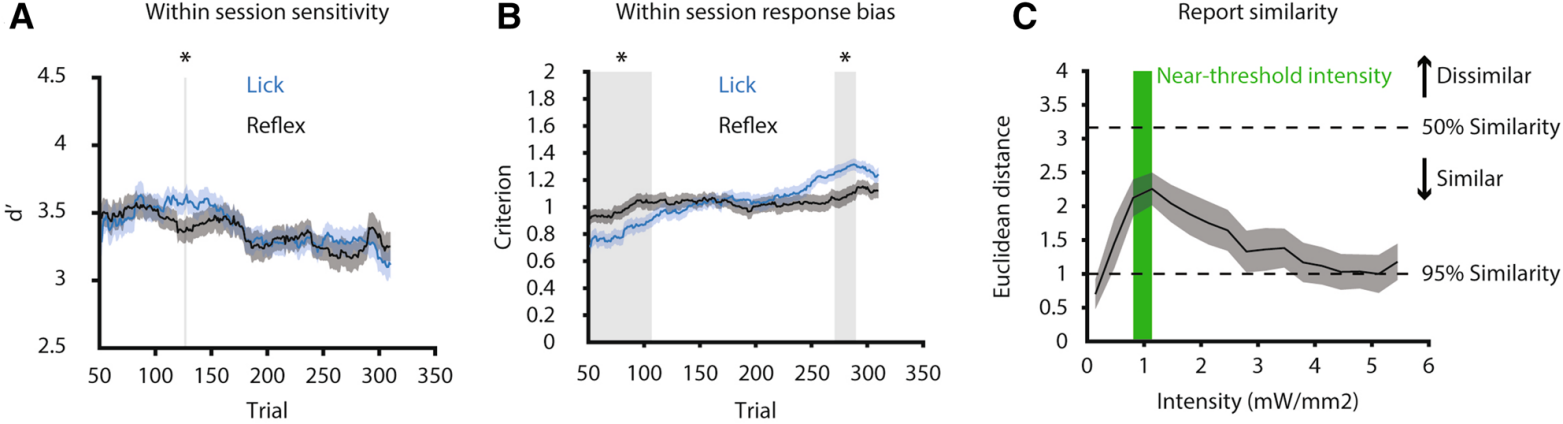
Withdrawal behavior and lick behavior become dissimilar near perceptual threshold compared to more salient stimuli. (**A**) Comparison of sensitivity, and (**B**) response bias as a function of time between lick versus withdrawal behaviors. These results show that sensitivity between behaviors is predominantly not significantly different within a session, while the criterion is significantly different between behaviors in the early and late stage of the session. This suggests that the decision to lick is influenced by higher-order processes that do not impact the reflex behavior. (**C**) Euclidean distance calculated as a function of stimulus intensity for lick versus withdrawal behavior. Green region indicates near-threshold intensities, while black dotted lines represent the Euclidean distance if response types were similar for 50% and 95% of all trials at any given intensity. Lick and reflex behaviors become dissimilar near threshold, which suggests that the behaviors are encoding different information. All results are shown as mean ± SEM, except for Euclidean distance, which is shown as mean ± 95% CI (n = 40), grey shaded regions and asterisks in a-c indicate significance at P < 0.025 using a two-sided Wilcoxon signed rank test. Data was plotted using MATLAB R2019a (https://www.mathworks.com). The figure was created using Adobe Illustrator CC 2019 (https://www.adobe.com).

To determine the effect of anti-nociceptive compounds on the lick behavior, we tested behavioral responses to 70 trials of a single light stimulus intensity (3.79 mW/mm^2^) following intraplantar injection of 2% lidocaine (n = 1). We found that while the mouse was able to self-report 97% of light stimuli following a control injection (0.9% normal saline), intraplantar injection of 2% lidocaine completely abolished the behavioral response.

### Lick-based, self-report uncovers unique behavioral response in pain perception

We then compared the lick behavior to hind quarter reflexes as opposed to hind paw withdrawal, as the paw was restrained for stimulation. We recorded video of the hind quarters during the lick behavior and scored stimulus-evoked reflex behaviors (Fig. 1F) that occurred within 250 ms of stimulus onset. Like the lick behavior, the hit rate of the reflex behavior was dependent on stimulus intensity and reached hit rates over 90% in response to optogenetic stimuli. When we compared the lick behavior with the reflex behavior at perceptual threshold, we found that not only were extrapolated threshold intensities (Supplemental Figure S1) and hit rates near perceptual threshold not significantly different (Fig. 2A), but hit rates for all intensities were not significantly different (P < 0.025, two-sided Wilcoxon signed rank, Bonferroni corrected). However, the lick behavior elicited a larger false alarm rate (30.9 ± 21.0%) compared to the reflex behavior (15.2 ± 17.2%, Fig. 2B). These findings suggest that the lick behavior, which is predominantly mediated by neural circuits in the brain, and the reflex behavior, which is predominantly mediated by neural circuits in the spinal cord, are comparable in their sensitivity for nociceptive detection, but diverge in their specificity.

To determine the probable cause for the differences in the performance between the lick and reflex behaviors across all sessions, we calculated the sensitivity (d’) and the response bias (criterion) metrics (Supplemental Figure S1). The sensitivity of the reflex behavior was significantly greater across all sessions than the sensitivity for the lick behavior (Fig. 2C). This difference was expected, as the reflex false alarm rate was significantly less than the lick false alarm rate. Criterion, however, was not significantly different between the two behaviors (Fig. 2C).

We then investigated whether the sensitivity and response bias differed between behaviors across time to determine if session averages masked any differences in performance. We calculated the d’ and criterion over a sliding 50-trial window within sessions. The sensitivity of both behaviors followed the same trajectory within sessions with little difference between behaviors (Fig. 3A). However, the lick criterion steadily increased as the reflex criterion remained constant (Supplemental Figure S1), thus accounting for significantly different criteria in the beginning and end of the sessions (Fig. 3B). Although additional investigation is necessary to determine the cause of this divergence, modulation of response bias has been attributed to the influence of top-down processes, such as motivation^34^, attention, and expectation, on perception and corresponding neural activity in the brain^35–37^.

Finally, we sought to determine whether the lick and reflex behaviors differed trial-by-trial. We hypothesized that if the lick and reflex provided redundant information, then the similarity between the behaviors should not be dependent on stimulus intensity. Therefore, we calculated the Euclidean distance between behaviors for each intensity across all sessions (Fig. 3C). We found that responses near perceptual threshold were significantly less similar (*P* < 0.025, two-sided Wilcoxon signed rank) compared to sub-threshold and supra-threshold responses. Therefore, the reflex behavior is less predictive of lick behavior when stimuli are perceptually ambiguous.

## Discussion

We have developed an assay whereby TRPV1-ChR2-EYFP transgenic mice rapidly and consistently self-report the detection of nociceptive stimuli over several hundred trials. Our lick behavior in this transgenic model is unique: it enables behavioral response quantification at sub-second timescales, it activates nocifensive afferents with millisecond precision, and it requires conscious decision-making by the subject in order to successfully perform the task. The behavioral assay is ideal for combined implementation with neural recording technologies to study the circuit level mechanisms and dynamics that give rise to pain perception.

The rationale for the development of our lick behavior was to overcome the issues inherent to the conventional paw withdrawal reflex; specifically, the inability to determine the involvement of brain circuits during pain processing. While licking may be considered an innate response to pain by which an animal tends to a site of injury^11^, the high degree of variability in performance within and across animals during training (Supplemental Figure S1) indicates that the spout-licking behavior is not a pre-programmed feature of the pain response, but a learned response to stimulus detection. This result is supported by previous findings that licking is not a direct or reliable sign of spontaneous neuropathic pain^38^. The significance of using a learned, self-reporting behavior instead of a spontaneous, reflex behavior is that the learned behavior can allow us to determine the effect that higher-order, cognitive processes have on the pain experience. Motivation and attention, for example, have been linked to the modulation of response biases during decision-making^34,35,37^, an effect that was exhibited within sessions by the lick behavior but not the reflex behavior. It is also important to acknowledge that a learned response to a noxious stimulus might not inherently reflect pain, but the association of a stimulus with a reward. Therefore, it is necessary to consider whether the lick behavior reflects pain or a novel stimulus response. In comparing the hit rates of the reflex behavior, which is a gold standard nocifensive metric, to the hit rates of the lick behavior across all sessions for each stimulus intensity, we found no statistically significant differences. This would suggest that on average the lick behavior is comparable to the reflex behavior for reporting pain. Interestingly, we found that on average the false alarm rate of the reflex behavior was significantly less than the false alarm rate of the lick behavior. While one interpretation of this result is that the reflex behavior has more specificity than the lick behavior for determining pain, the lick behavior false alarm rate was still significantly less than the hit rates near perceptual threshold. This would indicate that the lick behavior is still reliable for indicating pain. Furthermore, when comparing the two behaviors within sessions, we found that their sensitivities were not significantly different for most of the session (Fig. 3A). This suggests that the lick behavior could reflect the discriminative and the affective components of pain not captured by the reflex behavior.

Typically, aversion behaviors are employed to probe cognitive processes^11^. Operant conditioning in mice has been used to assess thermal pain tolerance by evaluating reward conflict in a conditioned place aversion (CPA) paradigm^39^. Although CPA and other operant methods enable the study of cognitive processes through non-innate, learned associations, these established aversive behaviors fail to capture the millisecond dynamics of decision-making. This results in the inability to directly map behavioral aversion to the neural activity that is explicitly linked to the perceived pain. The rapid self-report aspect of the detection assay provides more temporal precision in tracking behavioral responses driven by top-down control than is achievable by aversion behaviors.

Although the development and assessment of this behavior was focused specifically on evoked pain processing, the lick-based self-report has significant implications for the use of pain models, and the evaluation of pain therapies. Considerations should be given to future experiments when deciding on a specific pain model to avoid confounds in analyzing the lick-base self-report. Some pain models involving algogens, such as Formalin, can evoke prolonged bouts of spontaneous licking^11^ that could impact the lick-based self-report, while other compounds, such as zymosan, that have a dose-dependent response can elicit thermal or mechanical hyperalgesia without the presence of spontaneous pain behaviors^40,41^. Furthermore, neuropathic pain models that ligate or sever nerves of interest can be used to avoid spontaneous, non-evoked licking behavior^42^. In regards to pain therapies, analgesia is ordinarily determined by a shift in an animal’s pain threshold^42,43^. We found that an injection of lidocaine obliterated the self-report of a high intensity optogenetic stimulus, however, the type of analgesia and its concentration should have varying effects on self-reporting in this specific transgenic line. Furthermore, while we did not directly test our behavior with different pain models or other methods of analgesia, the divergence of responses between the lick and reflex behaviors near pain threshold suggest that reflex behaviors alone may not suffice as a measure of therapeutic outcome. This is further supported by the fact that reflex behaviors do not reflect affective components of pain^25^ that influence perception, which is a critical behavioral factor that determines therapeutic outcome.

While the cellular precision and specificity that optogenetics confers also adds to the potential merit of using this behavior for examining pain processing and pain therapies, it should be noted that the TRPV1-ChR2-EYFP transgenic line reported in this paper does show broad overlap (~ 50%) with DRG neurons^31^. A previous study that performed immunohistochemical analysis of this TRPV1 lineage line found that EYFP overlap predominately occurs in peptidergic and non-peptidergic nociceptors, and to a smaller degree non-noxious, myelinated neurons^29^. However, our electrophysiological characterization in these mice (Fig. 1B) suggests that our optogenetic approach is robust against recruitment of non-nociceptive primary afferents, judged by the absence of A alpha, and A beta volleys. We acknowledge that the transgenic line used in our experiments exhibits a unique optogenetic expression pattern that will only evoke a specific noxious percept, such as heat pain, and that it may not be a naturally occurring form of pain. However, the behavioral paradigm can easily be expanded to address other aspects of nociception by leveraging various genetic techniques. For example, inducible Cre-driver lines could provide developmental specificity in ChR2 expression^44^, while the implementation of other transgenic lines such as the SNS-ChR2^45^ and MrgD-ChR2^28^ could be used to investigate additional sub-populations of afferents that mediate other pain modalities. Such studies would be natural future extensions of the current work.

Although the use of peripheral optogenetics to evaluate pain behaviors has been previously established, our application of optogenetics in a detection paradigm to evoke self-reports of pain enables the acquisition of several hundred behavioral trials in a single session. Depending on the method used, paw withdrawal behaviors, such as von Frey, can take up to 2 hours and yield fewer than 100 trials^46^, which is a fraction of the total trials achieved during the lick behavior in double the time. This increase in behavioral output does come at a cost. One cost is that head fixation and hind paw restraint may affect the animal’s behavioral performance by inducing unnecessary stress and anxiety that does not occur in unrestrained behaviors. Although it has been shown that head and body restraint in rodents can increase stress-related corticosterone levels during behavioral tests involving painful stimuli, these corticosterone levels returned to baseline after several days of training^47^. Head and body restraint has also been shown to have no significant effect on mechanical and thermal withdrawal thresholds in rodents, however, a reduction in response to the formalin pain tests was also observed^48^. Reducing restraint-related stress and anxiety can be achieved through habituation and training^33,49, 50^. Importantly, these critical steps are also a cost of using the self-report behavior. While previous studies using head-fixation and hind paw restraint behaviors have achieved learning in as little as 4 days^18,19^, training on other head fixed behaviors can range from weeks to months depending on learning criterion and task difficulty^21,33,51^. Although some mice showed early success in training (Supplemental Fig. S1), our specific criterion resulted in training for roughly 10 sessions on average, or about 1.4 weeks at one session per day. Despite the longevity associated with learning, the ability of the animal to self-report greatly reduces the training burden of the researcher, who will only need to learn how to restrain the animal on the behavioral rig. In contrast, the demand for researcher involvement during standard evoked pain behaviors is large and problematic, given the inherent subjectivity of the researcher in performing the experiment^16^. Recent advances in machine learning have sought to remedy observer biases by automating scoring, and achieving sub-second resolution in the analysis of behavioral results^24^. Furthermore, machine learning has been used to classify “pain-like” and non-painful withdrawals behaviors, in an attempt to more accurately and objectively characterize rodent sensitivity to mechanical and thermal stimuli^26^. In conjunction with machine learning algorithms that characterize spinally-mediated pain behaviors, and chronic neural recordings, our self-report model can be used to dissect the higher-order brain circuits that give rise to pain, which would provide a much needed bridge between animal and human studies of pain and pain therapies.

## Materials and methods

### Transgenic mice

All methods were performed in accordance with the relevant guidelines and regulations and animal experiments were approved by the Institutional Animal Care and Use Committee at Rhode Island Hospital (RIH), Providence RI under protocol #5044-18. All mice, aged 2–24 months, were TRPV1-ChR2-EYFP, bred at RIH using the TRPV1-Cre and Ai32(RCL-ChR2(H134R)/EYFP) commercially available lines from Jackson Laboratories. Mice (n = 6) in acute electrophysiology experiments were half female and half male, mice used for behavior (n = 5) were male, and one female mouse was used for intraplantar injection and behavior. Mice were housed in groups of two or more, unless they received a headpost implant, in which case they were housed in single cages.

### Headpost surgeries

Mice were anesthetized using Isoflurane. Their heads were shaved and sterilized using betadine and 70% ethanol. Mice were then fixed in a stereotactic frame (Kopf instruments). An incision was made down the midline of the scalp and the skin was retracted. The exposed skull was then cleaned and scored to provide a dry surface for bonding with the dental cement. Titanium or stainless-steel head posts were then properly aligned and fixed to the skull using dental cement (MetaBond). Mice were then placed under a heating lamp or on a warming pad until they were bright, alert, and responsive. Behavioral testing and water restriction began no less than one week following implantation.

### Intraplantar hind paw injection

Following anesthetization with Isoflurane, a 20 µL intraplantar injection of either lidocaine (2%) or saline (0.9%) was given to the right hind paw. Experimental testing did not begin for 10–30 min following injection to ensure behavioral stability following anesthesia.

### Water restriction

Mice were acclimated over a five-day period to water restriction. Mice were kept at or above 85% of their pre-restriction baseline weight. Daily water volume was determined based on this criterion but could be no less than 1 mL/day. To determine that mice were kept at or above 85% of their baseline weight, weight and body conditioning score^52^ were taken a minimum of 3 days per week, and on everyday mice performed the detection behavior.

### Electrophysiological recordings from the spinal cord in anesthetized mice

TRPV1-ChR2-EYFP mice were anesthetized with isoflurane and shaved from their hips to below the base of their neck. A 2 cm incision was made on the surface of the back along the top of the spine. Following retraction of muscles along the spine, a laminectomy was performed on one of the vertebrae between T13 and L3. Once the spine was stabilized, a minimal durotomy was performed and a single epoxy coated, tungsten electrode (impedance 5MOhm, 127 µm diameter, A-M Systems) was lowered into the dorsal horn of the spinal cord using a micromanipulator arm. General receptive fields were explored first by tactile stimulation (brush) of the hind paw plantar surface, and then followed by brief light pulses (~ 1 s, 21mW/ mm^2^) generated by a 470 nm LED source (ThorLabs), thus providing optogenetic stimulation to the hind paw; stimuli were delivered at a 10 ms pulse width, 21mW/mm^2^, and 10 s inter stimulus intervals (ISI) for 100 trials.

Electrophysiological data were amplified and bandpass filtered at 0.1–3000 Hz using a WPI ISO-DAM8 and acquired by a CED Micro1401 (Cambridge Electronic Design, UK) data acquisition system. Signals were sampled between 12–34 kHz. Time stamps of optogenetic stimulation were registered by the data acquisition system using TTL logic.

### Histology and Immunohistochemistry

Following electrophysiological recordings and behavioral testing, spinal cord tissue was collected and placed in 4% formaldehyde in 1 × PBS overnight in 4 °C. Tissue samples were then placed in 30% sucrose in 1 × PBS for 3 days, until samples sank. Samples were then frozen in OCT using a mixture of dry ice and 70% ethanol. A cryostat was used to cut 25 µm sections of the spinal cord, and either stored in − 80 °C or mounted on slides immediately for staining. Slides were washed in 1 × PBS, three times for 10 min and then incubated with primary antibody (1:250 Rabbit anti-CGRP, Millipore-Sigma, PC205L) in blocking buffer solution (1 × PBS, 1% Bovine Serum Albumin, 0.1% Triton X-100) in 4 °C for 24 h. After primary antibody incubation, samples were washed with 1 × PBS three times for 10 min and then incubated for one hour with secondary antibody (1:500 Goat anti-Rabbit Alexa Fluor 635, ThermoFisher, A-31577) in blocking buffer solution at room temperature. Samples were then washed three more times in 1 × PBS for 10 min prior to mounting with Fluoromount-G (Thermo Fisher Scientific).

### Custom-built system for behavioral testing

To restrain the mouse hind paw, a custom platform was designed, and 3D printed. The platform features three M6 counter-bore holes to fix the platform to an aluminum breadboard (Thorlabs, MB4560/M) and a grated floor (5 × 5 grid of 4mmx4mm holes with 1 mm spacing) through which optogenetic and tactile stimuli were delivered (Supplemental Figure S1). Stimulation was delivered through the central hole in the grated floor via a ferrule attached to a fiber optic cable (Thorlabs, M98L01). Black hardboard was used to block horizontal stray light generated by the LED from the animal’s field of view, which is critical so that the animal is not alerted to visual cues from the light stimulation. Optogenetic stimulation was delivered using a 470 nm LED source (Thorlabs, M470F3) controlled by a LED driver (Thorlabs, LEDD1B) that received a modulatory analog input. Light tactile stimuli were delivered by attaching the fiber optic cable to a piezo-electric plate bender (Noliac, CMBP09) that was actuated with a piezo haptic driver (Texas Instruments, DRV2667). All aspects of the behavior were controlled by a Beaglebone black development board.

### Pain detection task

All mice were habituated for a minimum of 3 days (building from 15–60 min) without optogenetic or tactile stimulation to the behavioral platform, but with hind paw and head restraint using techniques adopted from a similar restraint methodology^18^. On the first day, mice were head-restrained for 15 min. On the second day, mice were head-restrained and their right hind paw was restrained using two layers of flexible medical tape and three layers of black masking tape for 30 min. The medical tape was positioned directly over the mouse’s paw to allow for comfort, and the masking tape was placed overtop the medical tape to block vertical stray light generated by the LED. On the third day, mice were head-restrained and hind paw restrained for 1 h. Following habituation to water restriction (5 days maximum), mice were then introduced to the lick spout in the behavioral system. Once mice displayed a persistent desire to lick over the course of 1 h, training on the detection behavior began. All sessions (training and testing) consisted of 360 trials that were randomly distributed to ensure that the trial order did not affect the animal’s response; 20 trials per each of the 17 target, optogenetic stimuli (10 ms pulse width, 0.14–5.45 mW/mm^2^) and a single catch, tactile stimulus (one cycle of ~ 120 Hz sine wave). A ceramic ferrule attached to a fiber optic cable and piezoelectric bender provided optogenetic (target) and tactile (catch) stimulation to the hind paw through the grated floor. Each trial had randomly distributed ISIs (between 3-6 s). Response windows were randomized between 0.8–1.5 s for each training session to ensure that mice could not predict when reward was being delivered during learning. Testing sessions maintained a 1 s response window. Additionally, each subsequent day of training the chance for receiving a water reward (5–10 µL) on a hit trial was reduced by 5–10% until the reward chance reached 80%, which was the reward chance for all behavioral testing sessions. This was done to maintain motivation during training by ensuring mice did not become sated too rapidly so that they could perform several hundred trials. During target trials, licking within the response window was considered a hit, giving mice an 80–100% (depending on the animal’s stage in behavior) chance of receiving a water reward (5-10uL). Not licking during the response window was considered a miss, and mice received up to 10 additional stimuli (10 ms pulse width, 100 ms ISI). If mice licked following a catch stimulus (false alarm), they received up to 10 ‘warning’ light pulses (10 ms pulse width, 100 ms ISI, 5.45 mW/mm^2^). Mice that licked prior to trial onset were given a time-out that lasted the length of the initial ISI (3–6 s). All behavioral experiments were performed using a Beaglebone Black Rev. C, linux computer, written in custom Python scripts (Version 2.7).

### Video capture

Video data were acquired at either 60 or 120 fps, 1080p using an offline GoPro Hero6 Black and stored as mp4 files directly onto a microSD card. Triggers were recorded as analog input through the on-board audio channel and later extracted from the corresponding mp4 file in MATLAB.

### Electrophysiological data analysis

Data were analyzed in MATLAB using available packages and custom scripts. Electrophysiological signals were bandpassed (0.1-100 Hz) and notch-filtered using EEGLAB 9.0^53^ before data were down-sampled to 8.3 kHz. Data were then epoched with respect to the stimulus onset and averaged for each mouse. Finally, data were normalized to the absolute maximal response in the averaged epoch.

### Behavioral data analysis

The sensitivity (d’) was used as a metric to determine which behavioral sessions were used for further analysis. Calculation of d’ was performed as previously described^21^ for detection behaviors in mice. For each session considered, d’ was calculated in a 50-trial sliding window, every trial using the equation:

$$Z\left( {hit\;rate - offset} \right) - Z\left( {false \;alarm rate + offset} \right)$$

where Z indicates the inverse of the Gaussian distribution, and the offset is set to 0.001 to avoid saturation of d’. Criterion was likewise calculated, using the equation:

$$\frac{{ - \left( {Z\left( {hit\; rate - offset} \right) + Z\left( {false\; alarm rate + offset} \right)} \right)}}{2}$$

Psychometric responses from lick behavior and reflex behavior were fitted with a cumulative Gaussian function using the MATLAB toolbox psignifit 4^54^. Fitting was performed only on optogenetic stimulation trials, while tactile trials were shown as a fixed rate. Euclidean distance was calculated using the following equation:

$$\surd \left( {\mathop \sum \limits_{i = 1}^{N} \left( {lick\; report_{i} - reflex \;report_{i} } \right)^{2} } \right)$$

Euclidean distance was calculated for each stimulus intensity for each session (20 trials per intensity). 95% and 50% similarity were determined by calculating the Euclidean distance for 19 and 10 trials, respectively, out of a total of 20 trials.

### Video data analysis

Video data were distributed among 3 observers who were blinded to the animal’s behavior, session number, and trial type. Observers were instructed to score according to a 0–3 scale comparing pre-stimulus to post-stimulus movement, and criterion for scoring were decided prior to scoring. Scores of “0” indicated no change in movement. Scores of “1” indicated subtle movement, such as a shift in weight on the hind quarters, but no lifting of the hind quarters. Scores of “2” indicated a mild response where the mouse’s hind quarters raised a small amount, with slow movement. Scores of “3” indicated an overt response where movements were immediate and rapid, with tail flicking and/or lifting of the hind quarters. Scores were then categorized as a hit or miss by grouping “2” and “3” scores, and “0” and “1” scores, respectively.

A custom MATLAB script was used to epoch each video trial to 250 ms pre-stimulus to 500 ms post-stimulus. Observers were trained on a practice dataset (37 trials from a later discarded session consisting of all trial types) prior to scoring behavioral data included in the analysis.

### Statistical analyses

All statistical analyses were performed using MATLAB R2019a. Data were tested for normality using a one-sample Kolmogorov–Smirnov test prior to any comparison. In all comparisons between lick and reflex behaviors (n = 40 sessions), as well as comparisons made with the false alarm rates, significance was determined using a two-sided Wilcoxon signed rank test with *P* < 0.025. Multiple comparisons were performed using the Bonferroni correction. A one-sided Mann–Whitney U test was used to compare differences in overall hit rate across sessions between control (n = 12) and test sessions (n = 28), with a significance level of *P* < 0.05.

## Supplementary information

Supplementary Figures.

## Data Availability

Custom components developed for the behavior are available for download on github (https://github.com/neuromotion/nociceptive-detection). The data that support the findings of this study are available on request from the corresponding author.
