# Supplementary material for: Automated and rapid self-report of nociception in transgenic mice
Source: Sci Rep. 2020 Aug 6;10:13215. doi: 10.1038/s41598-020-70028-8 (PMC7413385; doi:10.1038/s41598-020-70028-8)
Supplement: Supplementary file 1 — Supplementary Figures. [file 41598_2020_70028_MOESM1_ESM.docx]

**Supplementary Information**

**Automated and rapid self-report of nociception in transgenic mice.**

Christopher J. Black^1^, Anusha B. Allawala^1^, Kiernan Bloye^2^, Kevin N. Vanent^2^, Muhammad M. Edhi^3^, Carl Y. Saab^2,3,4^, David A. Borton^1,4,5,*^.

^1^School of Engineering, Brown University, Providence, RI, 02912, USA.

^2^Department of Neuroscience, Brown University, Providence, RI, 02912, USA.

^3^Department of Neurosurgery, Rhode Island Hospital, Providence, RI, 02903, USA.

^4^Carney Institute for Brain Science, Brown University, Providence, RI, 02912, USA.

^5^Center for Neurorestoration and Neurotechnology, Rehabilitation R&D Service, Department of VA Medical Center, Providence, RI, United States of America

*For correspondence: [daborton@brown.edu](mailto:david_borton@brown.edu) (D.A.B.)

**Supplementary Figures**

**
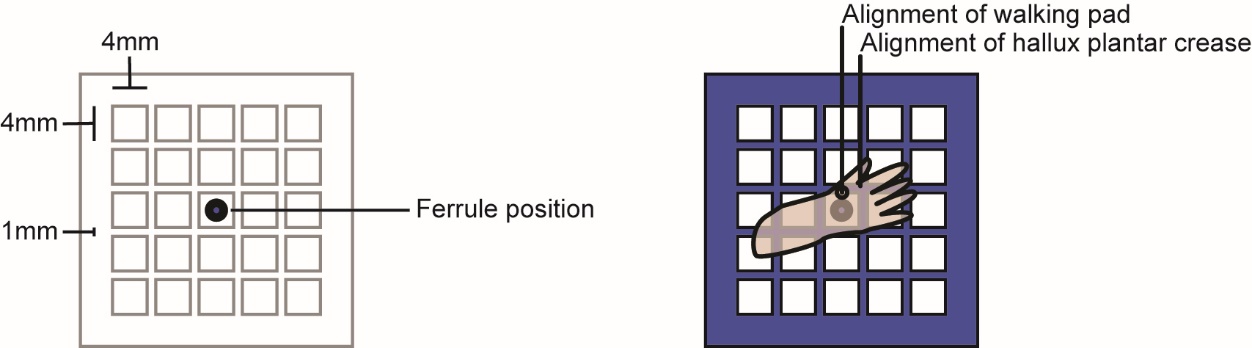
**

**Supplementary Figure 1.** Alignment of right hind paw over grated floor. (Left) 5x5 (4mm x 4mm holes with 1mm spacing) hole grated floor used for delivery of sensory stimuli. (Right) Crease between the first and second digit on the left is aligned with respect to the corner square containing the ceramic ferrule. Walking pads closest to the side of the foot were also used to position the hind paw in place. The figure was created using Adobe Illustrator CC 2019 (https://www.adobe.com).

**
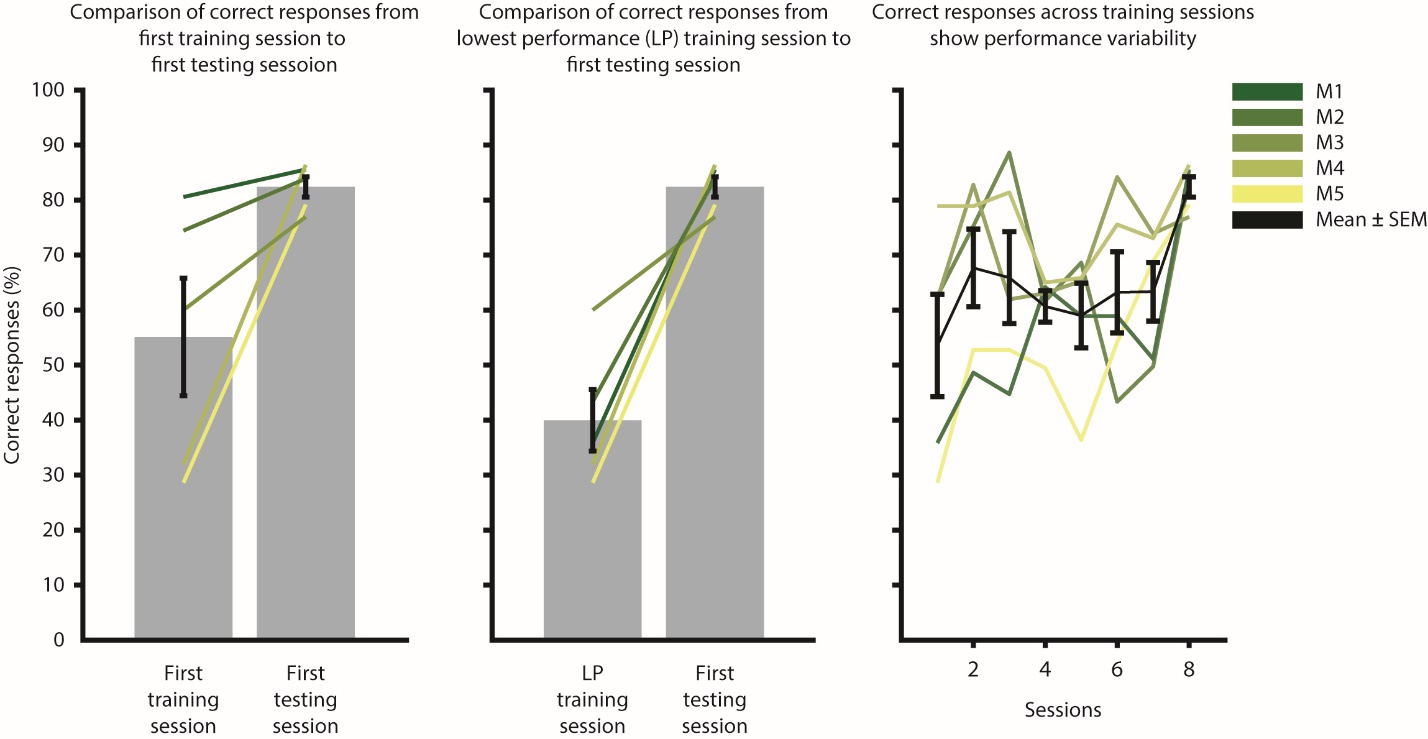
**

**Supplementary Figure 2.** Training variability shows the lick report is a learned behavior. (Left) Comparison between correct responses on the first training session with the correct responses on the first testing session. Mice display different rates of success on the first day of training. (Center) Mice with high initial performance in training (>65%) displayed dips in performance (<50%) in subsequent training sessions. (Right) Performance across mice varied in training sessions (sessions 1-7) leading to behavioral testing (session 8). Variability across training sessions indicate that the lick behavior is a decision. n = 5 sessions from each mouse, mean±SEM. Data was plotted using MATLAB R2019a (https://www.mathworks.com). The figure was created using Adobe Illustrator CC 2019 (https://www.adobe.com).

**
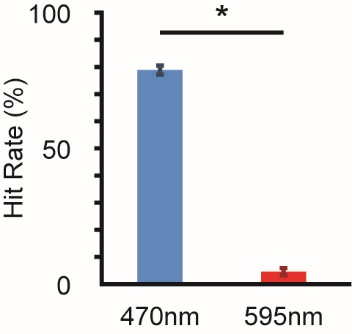
**

**Supplemental Figure 3.** Comparison of behavioral and control hit rate support that the self-report is driven by optogenetic activation of ChR2 in the periphery. Average hit rate of 3 mice for behavioral 470nm LED (blue, n = 28 sessions) and control 590nm control LED (red, n = 12 sessions), mean±SEM. Significant difference indicates that the thermal activation from the LED on the hind paw does not elicit the lick behavior. Significance of P < 0.05 using the Mann-Whitney U test. Data was plotted using MATLAB R2019a (https://www.mathworks.com). The figure was created using Adobe Illustrator CC 2019 (https://www.adobe.com).

**
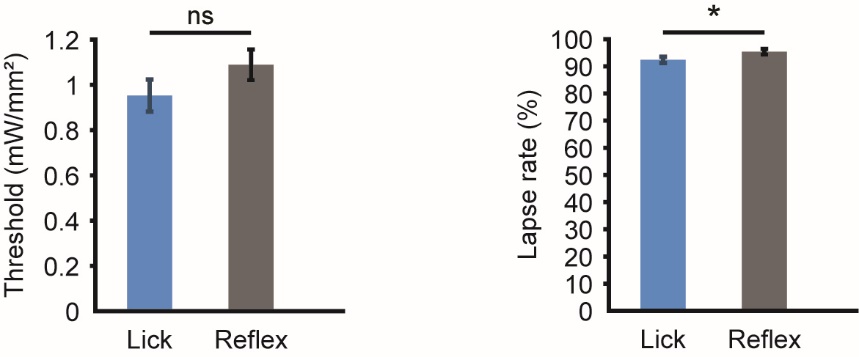
**

**Supplemental Figure 4.** Comparison of extrapolated threshold and lapse rates. Using the psignifit Matlab toolbox, the extrapolated threshold (left) for lick (blue) and reflex (grey) show no statistical difference; however, the extrapolated lapse rate (right) between lick (blue) and reflex (grey) are significantly different. These results show that on average, the lick and reflex behavior are comparable measurements of nociceptive threshold, but that the asymptotic limit of the behavioral response is greater for the reflex. n = 40 sessions from 5 mice, plotting mean±SEM, significance of P < 0.025 using the Wilcoxon signed rank test, multiple comparisons performed using the Bonferroni correction. Data was plotted using MATLAB R2019a (https://www.mathworks.com). The figure was created using Adobe Illustrator CC 2019 (https://www.adobe.com).

**
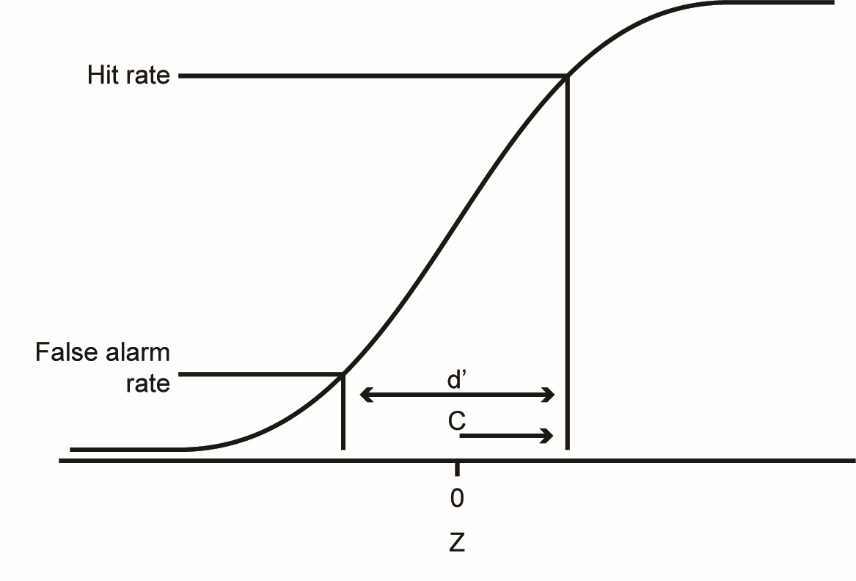
**

**Supplemental Figure 5.** Illustration of sensitivity (d’) and response bias (C) measures. While d’ is the difference between the signal and the signal + noise, it can be estimated by the difference of the inverse cumulative distribution function between the hit and the false alarm rates, whereas C can be estimated as one-half the sum of the inverse cumulative distribution function between the hit and false alarm rates. C will always be greater than 0, as 0 indicates the animals response biased to always reporting a stimulus. The figure was created using Adobe Illustrator CC 2019 (https://www.adobe.com).

**
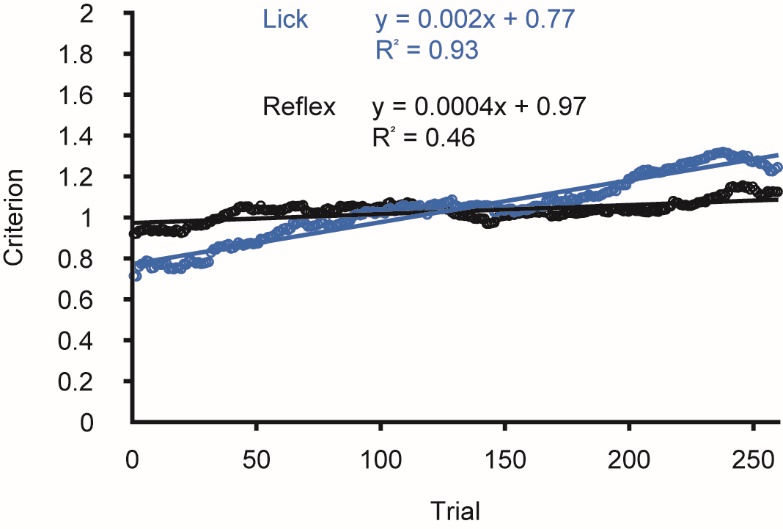
**

**Supplemental Figure 6.** Divergence in report criterion within sessions. Linear regression of both lick and reflex criterion. While both fits have a positive slope, the lick criterion (blue) has both a greater slope and R² than the reflex criterion (black), indicated a larger modulation of lick criterion over time (n = 40). Data was plotted using MATLAB R2019a (https://www.mathworks.com). The figure was created using Adobe Illustrator CC 2019 (https://www.adobe.com).
